# Synergetic Use of Sentinel-1 and Sentinel-2 Data for Soil Moisture Mapping at 100 m Resolution

**DOI:** 10.3390/s17091966

**Published:** 2017-08-26

**Authors:** Qi Gao, Mehrez Zribi, Maria Jose Escorihuela, Nicolas Baghdadi

**Affiliations:** 1 CESBIO (CNRS/CNES/UPS/IRD), 18 av. Edouard Belin, bpi 2801, 31401 Toulouse CEDEX9, France; mehrez.zribi@cesbio.cnes.fr; 2 isardSAT, Parc Tecnològic Barcelona Activa, Carrer de Marie Curie, 8, 08042 Barcelona, Spain; mj.escorihuela@isardsat.cat; 3 Observatori de l’Ebre (OE), Ramon Llull University, C.\ Horta Alta, 38, 43520 Roquetes, Spain; 4 IRSTEA, UMR TETIS, 500 rue Franois Breton, 34093 Montpellier CEDEX 5, France; nicolas.baghdadi@teledetection.fr

**Keywords:** soil moisture, SAR, Sentinel-1, NDVI, Sentinel-2, change detection

## Abstract

The recent deployment of ESA’s Sentinel operational satellites has established a new paradigm for remote sensing applications. In this context, Sentinel-1 radar images have made it possible to retrieve surface soil moisture with a high spatial and temporal resolution. This paper presents two methodologies for the retrieval of soil moisture from remotely-sensed SAR images, with a spatial resolution of 100 m. These algorithms are based on the interpretation of Sentinel-1 data recorded in the VV polarization, which is combined with Sentinel-2 optical data for the analysis of vegetation effects over a site in Urgell (Catalunya, Spain). The first algorithm has already been applied to observations in West Africa by Zribi et al., 2008, using low spatial resolution ERS scatterometer data, and is based on change detection approach. In the present study, this approach is applied to Sentinel-1 data and optimizes the inversion process by taking advantage of the high repeat frequency of the Sentinel observations. The second algorithm relies on a new method, based on the difference between backscattered Sentinel-1 radar signals observed on two consecutive days, expressed as a function of NDVI optical index. Both methods are applied to almost 1.5 years of satellite data (July 2015–November 2016), and are validated using field data acquired at a study site. This leads to an RMS error in volumetric moisture of approximately 0.087 m^3^/m^3^ and 0.059 m^3^/m^3^ for the first and second methods, respectively. No site calibrations are needed with these techniques, and they can be applied to any vegetation-covered area for which time series of SAR data have been recorded.

## 1. Introduction

Surface soil moisture plays an essential role in numerous environmental studies related to hydrology, meteorology and agriculture. For hydrological and agricultural applications, accurate soil moisture estimations are essential, since the hydric state of the soil is a key variable in the rainfall-runoff process [1]. Regular evaluation of this parameter can significantly improve flood and drought estimations [2], since it affects the amount of water available for vegetation growth [3,4]. In situ networks represent single point locations, and usually cover relatively short periods of observation [5], whereas the acquisitions of satellite data make it possible to continuously retrieve surface soil moisture, at regional and global scales. Various approaches have been developed for the retrieval of soil moisture, using optical, thermal infrared (TIR), and microwave (MW) sensors [6,7]. Optical sensors in the thermal spectrum are able to identify temperature differences, which can be related to surface soil moisture. Microwave soil moisture estimations are based on the strong contrast between the dielectric properties of water (≈80) and dry soil (<5) [8]. The depth at which the moisture is sensed depends on the sensor frequency but usually does not exceed several centimeters, in order to access rootzone soil moisture a more or less complex model is needed and several approaches have been developed such as techniques based on the energy balance approach based on thermal infrared soil moisture [9] or simplified water balanced approaches [10,11].

Traditional passive remote sensing instruments can be used to determine the surface soil moisture with a temporal resolution of 2–3 days. However, these instruments, which include the European Space Agency (ESA) Soil Moisture and Ocean Salinity (SMOS) mission [12,13] and the National Aeronautics and Space Administration (NASA) Soil Moisture Active Passive (SMAP) mission [14], have a low spatial resolution (around 40 km [15]). With the current Sentinel-1 mission, the active onboard C-band sensor offers regular temporal coverage (about five days for Europe when both A and B satellites are considered), together with a spatial resolution of 10 m.

In recent decades, SAR imagery has been shown to be advantageous for the estimation of soil surface characteristics, in particular surface roughness and soil moisture [16,17,18,19,20,21,22,23]. SAR data in the L, C and X bands is widely used for soil moisture retrieval [15,16,19,21,22,23,24,25,26,27,28,29,30,31,32,33,34], and the C-band sensor carried by Sentinel-1 has demonstrated its ability to retrieve soil characteristics over vegetation-covered surfaces [35,36,37,38,39,40,41,42]. The Sentinel-1 data can either be used to retrieve soil moisture or for downscaling SMOS or SMAP soil moisture. By using active and passive microwave data fusion method [43], it could be possible to retrieve soil moisture at a higher accuracy.

Radar remote sensing measurements of bare soil are very sensitive to the surface-layer water content, due to a pronounced increase in the dielectric constant of the soil with increasing water content [43]. In the last twenty years, different empirical, semi- empirical and physical models have been proposed for the retrieval of soil moisture from various sources of SAR data (ERS, RADARSAT, ENVISAT, TerraSAR-X, etc.). At the field scale, inversion models often take into account the effects of roughness and vegetation, due to their significant influence on radar signals. The most widely used techniques for the retrieval of soil moisture from SAR data include the Neural Network (NN) approach [17,44,45,46,47,48,49,50,51,52,53,54], the Water Cloud Model (WCM) [39,41,55,56,57,58,59], and the Change Detection method (CD) [11,60,61,62,63,64,65,66,67,68,69].

The artificial NN technique involves nonlinear parameterized mapping from an input vector to an output vector [70,71]. Santi et al. [46] reported retrieved soil moisture measurements derived from ENVISAT/ASAR data, using an artificial neural network (ANN) technique. The neural network was trained using satellite backscattering coefficients and soil parameters measured during simultaneous ground-truth campaigns, characterized by an RMSE of 0.023 m^3^/m^3^. Baghdadi et al. [52] retrieved soil moisture values from C-band SAR data using the NN technique, with an RMSE close to 0.098 m^3^/m^3^ in the absence of a priori information related to the soil parameters, and an RMSE of 0.065 m^3^/m^3^ when a priori soil moisture data obtained over bare agricultural areas was included in the analysis.

The Water Cloud Model (WCM) approach can be used over densely vegetated areas, since it relates the backscattering coefficient to soil moisture content and the presence of vegetation. He et al. [57] estimated the soil moisture of an alpine grassland area, using the Integral Equation Method (IEM) and the modified Water Cloud Model (WCM), leading to R^2^ = 0.71 and RMSE = 0.0332 m^3^/m^3^. Two-thirds of the data points derived from field surveys were used to parameterize the backscattering model, and the remainder were used for validation. Zribi et al. [41] estimated soil moisture values from C-band ASAR data using the WCM, leading to an RMSE of approximately 0.06 m^3^/m^3^ over a semi-arid region. Typical input vegetation parameters include the albedo of the vegetation and the attenuation factor, both of which are difficult to define. Laboratory-based measurements of the vegetation water content can be used when a high level of accuracy is required [72].

When multi-temporal SAR data is available, the Change Detection (CD) approach can be advantageously used, in the absence of prior knowledge of the study area. Zribi et al. [68] mapped soil moisture in a semi-arid region using ASAR/Wide Swath satellite data, based on the CD approach, with an RMSE equal to 0.13 (approximately 0.035 m^3^/m^3^ as volumetric moisture) over a semi-arid region. This approach makes the assumption that changes in vegetation and soil roughness have only a minor influence on variations in backscattering coefficient, which are dominated mainly by changes in the value of soil moisture [73].

In the present paper, two simple CD methodologies are applied, without field calibrations, and validated over an area characterized by a dense vegetation cover (irrigated crop fields). The soil moisture is retrieved from the synergetic interpretation of Sentinel-1 and Sentinel-2 data. The mean soil moisture is computed with a resolution of 100 m, which is compatible with agricultural applications. Despite S1 spatial resolution being around 10 m, the soil moisture is estimated a lower resolution (100 m) in order to decrease uncertainties caused by different types of heterogeneities in agricultural fields such as local changes in roughness, heterogeneities in vegetation cover etc. The first proposed method is based on the interpretation of backscattering statistics from Sentinel-1 observations, using the minimum and maximum values of this parameter throughout the full period of observation, whereas the second method is based on the analysis of backscattering differences on two consecutive acquisition days. With both methodologies, the Sentinel-1 data is combined with the normalized difference vegetation index (NDVI) computed from Sentinel-2 data. Site calibration is not mandatory for these two methods.

Our paper is organized as follows: in Section 2, the studied site and database are presented. Section 3 describes the two proposed methodologies. Section 4 presents our validation methodology, the ground measurements, and the resulting soil-moisture maps. Finally, our discussion and conclusions are presented in the last section.

## 2. Study Site and Database

### 2.1. Study Site

The study area covers a 20 km by 20 km area and is located in Urgell (Catalunya, Spain). The Urgell climate is typically Mediterranean, with a continental influence: mild in winter and warm in summer, with a very dry summer season and two rainier seasons in autumn and spring [74].

The average annual temperature lies between 13 and 14 °C for most of the region, where winter temperatures are low and the summer climate is rather warm, with frequent cool nights. The average yearly rainfall is less than 500 mm for most of the region, with the winters having low rainfalls, and the summers being very dry.

More than 80% of the Urgell area is cultivated and there is very little natural vegetation, except in the mountainous areas towards the northern and southern extremities of the region. The most common crops are corn, fruit trees, wheat and alfalfa. In the mountains, the natural vegetation consists of oak forests.

**Figure 1:**
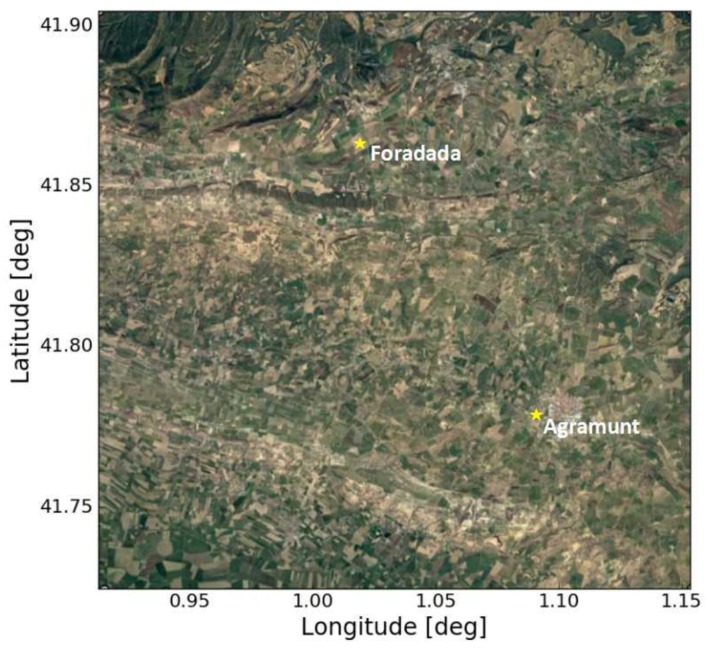
Study area in Urgell, Catalunya (20 km × 20 km) with ground measurements (yellow stars).

An old irrigated district located in this region has an open channel (built in 1862) transporting water towards the agricultural fields, thus allowing the vegetation to flourish in this specific area. Although the land surrounding the irrigated area is much drier, and a new irrigation network is being developed to augment the coverage of the old irrigation system, its influence is not yet visible on satellite imagery. The locations of two demonstration fields (inside the new irrigation area, at Foradada and Agramunt) are shown in Figure 1.

### 2.2. Database

#### 2.2.1. Ground Measurements

In-situ soil moisture measurements were acquired continuously (5 min sampling frequency) over a period of several months, in two demonstration fields belonging to the new irrigation district: Foradada and Agramunt (Figure 1). Each measurement point was analyzed at different depths. The precipitation data comes from the nearest meteorological station to the demonstration field. For Foradada field, the Baldomar station, which is about 6 km away, is taken, while for Agramunt field, the Tornabous station, which is about 11 km, is considered. Table 1 lists the measured soil moisture and texture characteristics of the two test fields.

#### 2.2.2. Satellite Data

(1) Sentinel-1 data

The Sentinel-1 satellites are equipped with C-band Synthetic Aperture Radar (SAR) instruments, providing data in dual or single polarizations. Sentinel-1 provides data with a spatial resolution of 10 m and a temporal resolution of 12 days, in both VV and VH polarizations. In the present study, signals recorded in the VV polarization were used to compute the soil moisture estimations. Only one ground track (110) was considered, for which the incidence angle was approximately 40.3°. Previous studies have shown that VH data has only a limited potential for the estimation of soil moisture, in particular as a consequence of its high sensitivity to volume scattering, which depends strongly on the geometrical alignment and characteristics of the vegetation [75,76,77]. The Sentinel-1 satellite database corresponds to the period from July 2015 to November 2016 (Table 2). All of the Sentinel-1 data was pre-processed using the Sentinel-1 Toolbox, in three steps:

- Thermal noise removal
- Radiometric calibration
- Terrain correction using SRTM DEM at 30 m.

The last step is needed to average the data over 100 m pixels or cells. As discussed above, the methodologies proposed in this study were developed on the basis of this spatial resolution, which also has the advantage of eliminating speckle effects in the radar data.

(2) Sentinel-2 data

The Sentinel-2A satellite was launched in June 2015, and was followed by Sentinel-2B in March 2017. It is a wide-swath, high-resolution, multi-spectral imaging mission, and is designed to provide full and systematic coverage of the Earth’s land surfaces [78]. The Sentinel-2 database corresponds to the period from July 2015 to November 2016 (Table 3). The Sentinel-2 data corresponds to images recorded in 13 spectral bands, with a spatial resolution of 10 m. In the present study, band 4 (Red) and band 8 (NIR) are used to calculate the NDVI:

(1) $$\mathrm{NDVI}=\frac{\left(\mathrm{NIR}-\mathrm{Red}\right)}{\left(\mathrm{NIR}+\mathrm{Red}\right)}$$

**Figure 2:**
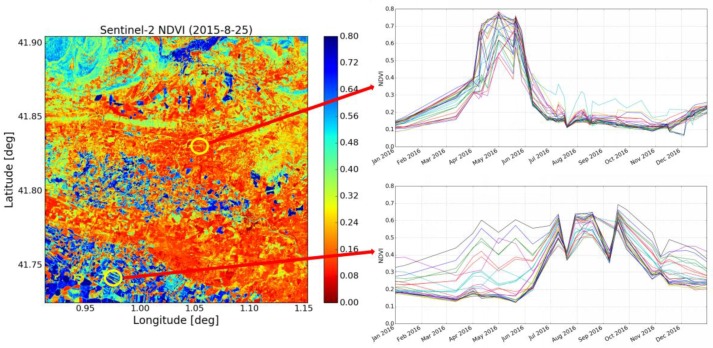
Sentinel-2 NDVI map in the study area (**left**), NDVI time series for the dry land site (**upper right**), and NDVI time series for the site in the old irrigated area (**lower right**).

Band “QA60”, which is a bit-mask band containing cloud mask information, is applied in order to remove areas covered by cloud. Figure 2 is a NDVI map of our study area, computed from Sentinel-2 data recorded on 25 August 2015, and characterized by a dynamic range between 0.1 and 0.8. The temporal variations in NDVI during 2016 are shown for two different locations:

- The first of these corresponds to dry (non-irrigated) land, revealing an NDVI cycle that occurs between April and July, with a low NDVI for the remaining periods of the year. This trend is confirmed for all of the pixels observed at this location.
- The second site corresponds to an irrigated area, which is characterized by a broad range of spatial and temporal variations in NDVI.

In order to develop suitable soil moisture algorithms, a mask is used to remove high density vegetation areas with an NDVI > 0.8, corresponding to forests that are not encountered in the agricultural pixels, and low density vegetation areas with an NDVI < 0.1, corresponding to water surfaces.

## 3. Proposed Methodologies

### 3.1. Method 1 Description

The first method involves retrieval of soil moisture using the radar signal CD technique. This approach to soil water content estimations has already been applied to data recorded by the ERS Scatterometer over West Africa [15]. In the present study, this method was adapted to the characteristics of the Sentinel-1 observations, and the inversion algorithm was optimized to take advantage of the high repeat rate of this data. The radar signals backscattered by the surface can be modeled as the sum of the radar signals scattered by the bare soil and attenuated by vegetation effects, and the signals scattered by the vegetation cover. These two contributions can be expressed as:

(2) $$\sigma_{\mathrm{cover}}^{0}=\sigma_{\mathrm{veg}}^{0}+\gamma^{2}(\theta )\sigma_{\mathrm{soil}}^{0}$$

where $\gamma^{2}\left(\theta \right)=\exp\left[-2\tau /\cos\left(\theta \right)\right]$ is the two-way vegetation canopy transmissivity, θ is the incidence angle and τ is the optical thickness parameter that depends on the type of geometrical structure and vegetation water content of the canopy [79].

Temporal variations in soil moisture can be directly related to the dynamics of the radar signal. When radar signals are considered for the same 100 × 100 m cell, and for approximately the same NDVI index, the roughness effect can be considerably reduced by computing the difference between two radar signals recorded at two dates.

For a given NDVI (retrieved from S2 data), by taking all of the corresponding radar data into account, the minimum value of σ^0^, corresponding to the driest signal, can be determined for each cell. The radar signal difference for a given cell (i,j), between one radar signal at date d and the driest signal, can be written as follows:

(3) $$\Delta \sigma_{{}_{\left(i,j\right)}}^{\mathrm{NDVI}}=\sigma_{(i,j),\mathrm{NDVI}}^{0}(d)-\sigma_{\mathrm{dry},(i,j),\mathrm{NDVI}}^{0}=H_{\left(i,j\right)}(\mathrm{NDVI},\mathrm{Mv})$$

where $\sigma_{(i,j),\mathrm{NDVI}}^{0}(d)$ is the backscattered signal from cell (i,j) at date d, with the corresponding NDVI computed from the (S2) optical images; $\sigma_{\mathrm{dry},(i,j),\mathrm{NDVI}}^{0}$ is the lowest backscattered signal, corresponding to the driest conditions, and computed using the S1 time-series using the same NDVI as for the data recorded on date d ($\sigma_{(i,j),\mathrm{NDVI}}^{0}(d)$), and $H_{\left(i,j\right)}(\mathrm{NDVI},\mathrm{Mv})$ is a function of the NDVI and soil moisture Mv in cell (i,j).

As our radar database covers a period of only 1.5 years (due to the later launch date of the Sentinel-2 satellite, i.e., June 2015), it was not possible to retrieve this relationship for each value of NDVI. We thus consider NDVI classes for the computation of $\sigma_{\mathrm{dry},(i,j),\mathrm{NDVI}}^{0}$, using intervals of 0.1 (0.1–0.2, 0.2–0.3, 0.3–0.4, etc.). In the present case, the NDVI over the studied agricultural site ranges between a minimum of 0.1 and a maximum of 0.8.

Various experimental studies have shown that a linear relationship exists between radar signal differences and changes in soil moisture [19,80], in the case of bare soils and vegetation-covered surfaces. For a given NDVI, the radar signal difference, $\Delta \sigma_{}^{\mathrm{NDVI}}$, can thus be written as:

(4) $$\Delta \sigma^{\mathrm{NDVI}}=\alpha (\mathrm{NDVI})\Delta \mathrm{Mv}$$

where ∆Mv is the change in soil moisture between the date d and the date when the soil was at its driest. The parameter α depends on the NDVI.

**Figure 3:**
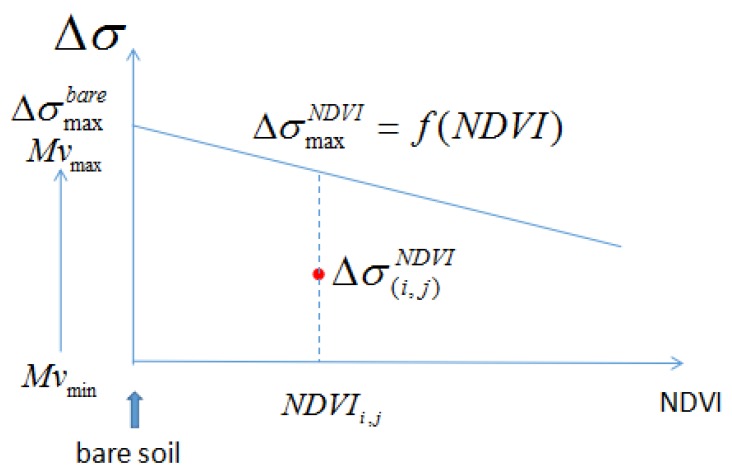
Illustration of the relationship between NDVI and Mv used in method 1.

When the NDVI increases, the moisture sensitivity of the signal can be expected to decrease [22,81], as shown in Figure 3. This means that the difference between surface backscattering at a given date d, and that observed on the driest date, decreases as a function of NDVI.

The strongest variation in moisture, $\Delta {\mathrm{Mv}}_{\max}$, corresponding to the difference between the driest value (Mv_min_) and the wettest conditions (Mv_max_), can be written as:

(5) $$\Delta {\mathrm{Mv}}_{\max}={\mathrm{Mv}}_{\max}-{\mathrm{Mv}}_{\min}$$

Under the conditions for which $\Delta {\mathrm{Mv}}_{\max}$ is defined, the maximum variation in backscattered signal (for a fixed value of NDVI), can be written as:

(6) $$\Delta \sigma_{\max}^{\mathrm{NDVI}}=\alpha (\mathrm{NDVI})\Delta {\mathrm{Mv}}_{\max}=f\left(\mathrm{NDVI}\right)$$

**Figure 4:**
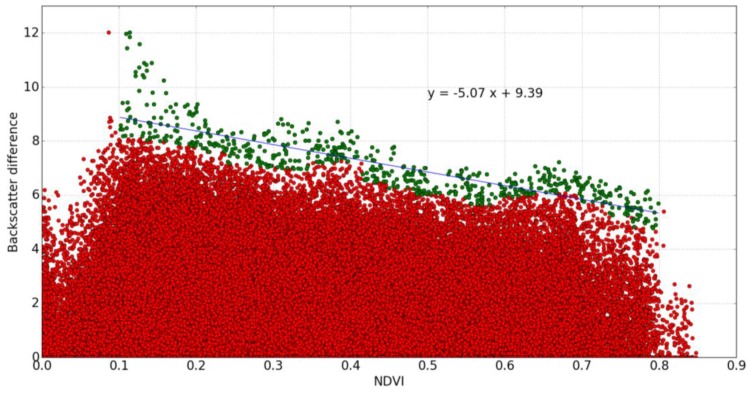
Illustration of the processed radar signal differences (dB) for all dates, with the driest radar signals shown as a function of NDVI for all (100 m × 100 m) cells in the Urgell area. Each point corresponds to a single radar signal difference $\Delta \sigma_{{}_{\left(i,j\right)}}^{\mathrm{NDVI}}$ for a cell (i,j).

The predicted values of backscattered signal difference, corresponding to S1 data, are shown as a function of NDVI in Figure 4. The backscattering difference calculations were carried out for all cells and all S1 acquisition dates (over a period of approximately two years).

$\Delta \sigma_{\max}^{\mathrm{NDVI}}$ is modeled as [15]:

(7) $$\Delta \sigma_{\max}^{\mathrm{NDVI}}=f\left(\mathrm{NDVI}\right)=a\;\mathrm{NDVI}+\Delta \sigma_{\max}^{\mathrm{bare}}$$

When NDVI = 0, $\Delta \sigma_{\max}^{\mathrm{NDVI}}=\Delta \sigma_{\max}^{\mathrm{bare}}$, which corresponds to the maximum value of backscattering difference under the driest, bare-soil conditions.

In order to minimize the influence of noise when estimating f(NDVI), for each selected value of NDVI, we excluded the upper 1% of the corresponding values of radar signal difference, as well as all data points having a radar signal lower than −15 dB, since these are known to correspond to water [46,82] (Figure 4).

The soil moisture for each pixel can thus be retrieved using the following function:

(8) $$\mathrm{Mv}\left(i,j,\mathrm{NDVI},d\right)=\frac{\Delta \sigma_{\left(i,j\right)}^{\mathrm{NDVI}}}{f\left(\mathrm{NDVI}\right)}\left({\mathrm{Mv}}_{\max}-{\mathrm{Mv}}_{\min}\right)+{\mathrm{Mv}}_{\min}\left(i,j,d\right)$$

**Figure 5:**
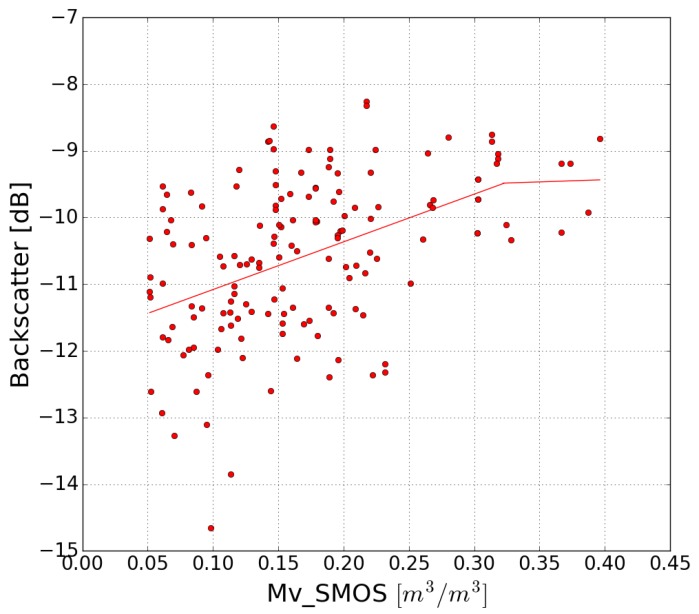
Mean S1 radar signal as a function of the SMOS soil moisture computed over a single SMOS pixel (40 km × 40 km). The radar signal saturates beyond soil moisture levels of 0.32 m^3^/m^3^.

SMOS low-resolution moisture products (SMOS Level 3 daily product), corresponding to the two-year period of S1 acquisitions, were used to estimate ${\mathrm{Mv}}_{\max}$ and ${\mathrm{Mv}}_{\min}$, since the ground measurements were recorded for a limited period of time. The mean S1 radar signal is estimated over a SMOS pixel (40 km × 40 km). Figure 5 plots the relationship between this mean radar signal and the SMOS moisture values, for dates that are common to both SMOS and S1 acquisitions. An approximately linear relationship is found between the values of volumetric soil moisture and the backscattered radar signal, up to Mvmax ≈ 0.32 m^3^/m^3^, following which it saturates with a constant radar signal strength of approximately −9.5 dB. This result confirms the findings of several scientific studies, which have revealed radar signal saturation for soil moisture levels in the range (0.3–0.35 m^3^/m^3^) [21,81]. From this result, when using method 1 we consider ${\mathrm{Mv}}_{\max}$ = 0.32 m^3^/m^3^. As shown shown in Figure 5, the value of ${\mathrm{Mv}}_{\min}$ is taken to be ≈ 0.05 m^3^/m^3^.

### 3.2. Method 2 Description

A second change detection approach is proposed in this paper. This is based on the difference in backscattered signals observed on two consecutive days of Sentinel-1 data (12 days). Under these conditions, the temporal change in vegetation cover is generally very small such that, for a nearly constant value of roughness and constant vegetation conditions, the difference between the backscattered signals depends mainly on the change in soil moisture [24].

**Figure 6:**
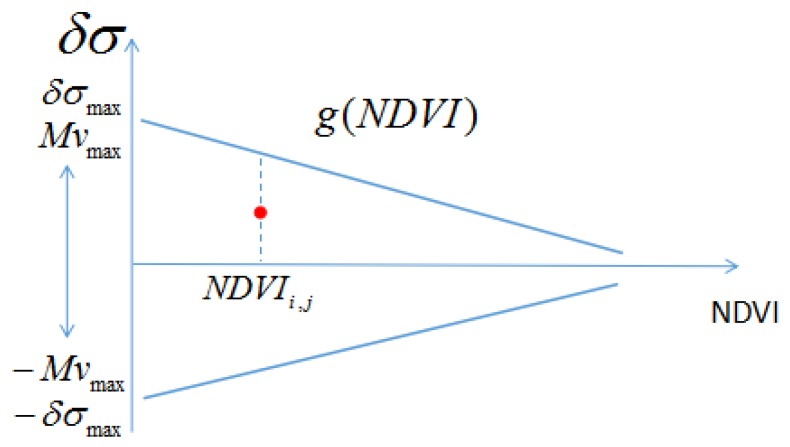
Illustration of method 2.

When the value of the NDVI increases, the radar signals’ sensitivity to temporal variations in moisture decreases. This means that the absolute value of the S1 radar signal difference decreases over two consecutive days, provided that the NDVI remains approximately stable on these two dates. In the present case, the latter parameter is taken to be the average of the NDVI values observed for the two consecutive dates. Figure 6 shows this change (either negative or positive) in radar signal behavior for successive S1 acquisitions, as a function of NDVI. $\delta \sigma_{\max}^{\mathrm{NDVI}}$ is the maximum change in radar signal, corresponding to the maximum value of soil moisture change $\delta {\mathrm{Mv}}_{\max}$, for a given value of NDVI. This can be modeled by the empirical function g:

$$\delta \sigma_{\max}^{\mathrm{NDVI}}=g\left(\mathrm{NDVI}\right)$$

**Figure 7:**
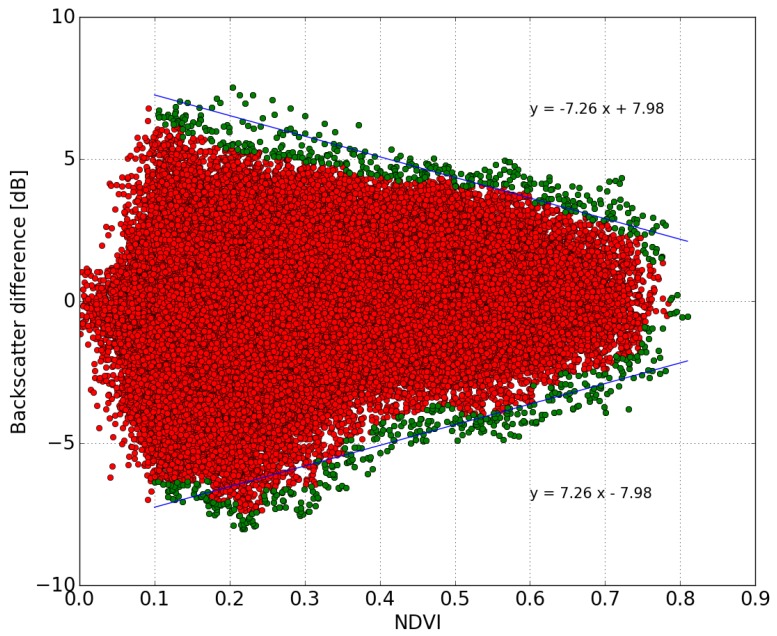
Illustration of the radar signal difference (dB) computed for two consecutive dates, as a function of NDVI over the Urgell site. Each point corresponds to a single cell (i,j). For each value of NDVI, the green points indicate the upper decile of the corresponding differences in radar signal.

Figure 7 shows the difference in radar signal between two consecutive dates, as a function of NDVI, for all cells (i,j) and all NDVI levels at the Urgell site. The radar signal difference (negative or positive) between two adjacent days decreases in absolute value, when the NDVI increases. The negative or positive radar signal differences, resulting from respectively increasing or decreasing values of soil moisture, can be seen to follow a symmetrical, linear pattern.

In the case of the maximum value of soil moisture change $\delta {\mathrm{Mv}}_{\max}$, the function g can be written as:

(9) $$\delta \sigma_{\max}^{\mathrm{NDVI}}=g\left(\mathrm{NDVI}\right)=b\;\mathrm{NDVI}+\delta \sigma_{\max}^{\mathrm{bare}}$$

where $\delta \sigma_{\max}^{\mathrm{bare}}$ is the maximum radar signal difference between two consecutive measurements over bare soil, associated with the highest value of moisture change.

When the NDVI is equal to zero, $\delta \sigma_{\max}^{\mathrm{NDVI}}$ is equal to $\delta \sigma_{\max}^{\mathrm{bare}}$, where b is the slope of the empirical function g. This describes the decrease in radar signal sensitivity to soil moisture. We observe an approximately symmetrical result in the computed values for the upper and lower limits. This is due to the fact that for a given value of mean soil moisture, a very similar behavior results from either a decrease or an increase in soil moisture, as these are linearly related to the radar signal.

In order to minimize the influence of noise arising from rare events, when estimating the function g(NDVI), for each selected value of NDVI we exclude the upper 1% of the corresponding values.

For a given NDVI, the backscatter difference $\delta \sigma \left(t_{1},t_{2}\right)$, with t_1_ and t_2_ being adjacent S1 acquisition dates, is assumed to be linearly correlated with the soil moisture difference. The soil moisture difference $\delta \mathrm{Mv}\left(t_{1},t_{2}\right)$ for each cell (i,j), between successive acquisition dates t_1_ and t_2_, can be retrieved using the following function:

(10) $$\mathrm{Mv}\left(i,j,t_{1}\right)=H\left(\delta \sigma \left(t_{1},t_{2}\right)\right)+\mathrm{Mv}\left(i,j,t_{1}\right)$$

where H is equal to:

$$H\left(\delta \sigma \left(t,t+1\right)\right)=\frac{\delta \sigma_{\mathrm{NDVI}}}{g\left(\mathrm{NDVI}\right)}\left(\delta {\mathrm{Mv}}_{\max}\right)$$

From the ground measurement statistics, the maximum soil moisture difference between two adjacent dates of Sentinel-1 data, $\delta {\mathrm{Mv}}_{\max}$, is assumed to be equal to 0.15 m^3^/m^3^.

From a starting date t_1_, which in the present case is a date corresponding to a ground measurement, an iterative calculation is used to determine the soil moisture for the following dates t_1_, t_2_, t_3_, …:

(11) $$\mathrm{Mv}\left(i,j,t_{2}\right)=\mathrm{Mv}\left(i,j,t_{1}\right)+H\left(\delta \sigma \left(t_{1},t_{2}\right)\right)\;\mathrm{Mv}\left(i,j,t_{3}\right)=\mathrm{Mv}\left(i,j,t_{2}\right)+H\left(\delta \sigma \left(t_{2},t_{3}\right)\right)\;......$$

## 4. Results and Discussion

### 4.1. Results

Using ground measurements recorded in the Foradada field from May to August 2015, and from February to October 2016, and in the Agramunt field from May to October 2015, and from July to November 2016, the values of retrieved soil moisture were validated with Sentinel-1 data, using the two approaches described in the previous section. We compare the satellite estimations with surface moisture measurements obtained at a depth of 3 cm in the Foradada field, and at a depth of 5 cm in the Agramunt field.

#### 4.1.1. Method 1 Validation with Ground Measurements

**Figure 8:**
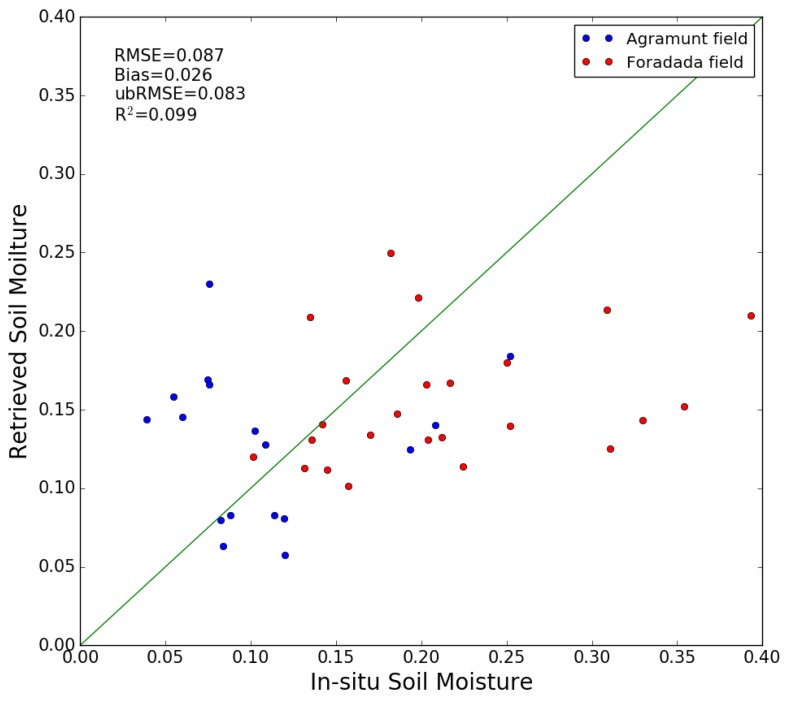
Intercomparison between ground measurements and S1 moisture estimations based on method 1, for the case of two demonstration fields, at Agramunt and Foradada.

Figure 8 compares the ground measurements with the values of soil moisture modeled using method 1. The Root Mean Square (RMS) error in volumetric soil moisture is 0.087 m^3^/m^3^, with a bias of approximately 0.026 m^3^/m^3^. For Agramunt field, the RMSE is 0.074 m^3^/m^3^, with a bias of −0.019 m^3^/m^3^ and for Foradada field, the RMSE is 0.095 m^3^/m^3^, with a bias of 0.057 m^3^/m^3^. The RMSE can be estimated more reliably by defining an unbiased RMSE [83]:

(12) $$\mathrm{ubRMSE}=\sqrt{E\{{\left[({\mathrm{Mv}}_{\mathrm{retrieved}}-E[{\mathrm{Mv}}_{\mathrm{retrieved}}])-({\mathrm{Mv}}_{\mathrm{insitu}}-E[{\mathrm{Mv}}_{\mathrm{insitu}}])\right]}^{2}\}}$$

where E[·] is the expectation operator. The unbiased RMSE corresponding to the first method is 0.083 m^3^/m^3^, which is equal to 0.071 m^3^/m^3^ for Agramunt field, and to 0.076 m^3^/m^3^ for Foradada field.

**Figure 9:**
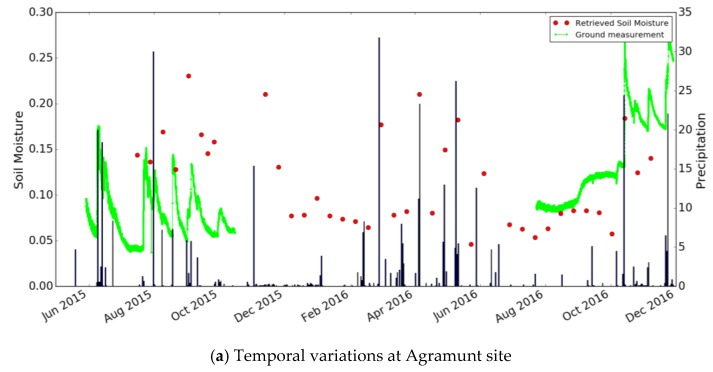

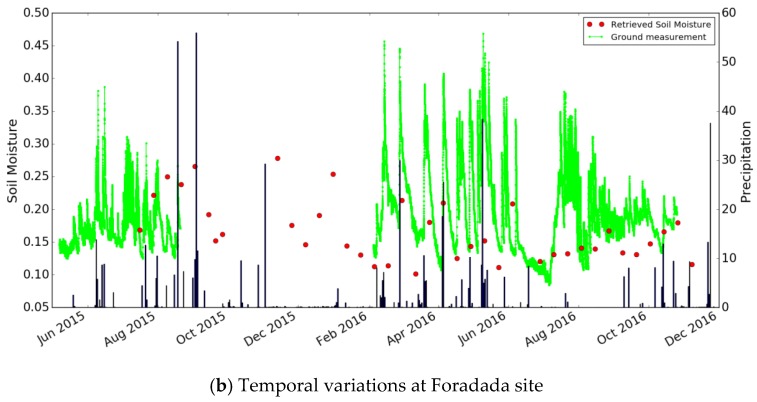
Temporal variations in ground measurements and S1 estimations of soil moisture at the Agramunt site (**a**) and Foradada site (**b**).

It can be seen that the errors are particularly high in the case of high moisture levels. This is due to possible variations in saturation moisture levels, and/or to spatial variations in soil roughness at the studied site. The statistical analysis should be improved by using a larger number of data acquisitions from the S1 time series. This can be expected to improve calibration of the function f. Figure 9 compares the soil moisture estimations with the ground measurements, as a function of time. The soil moisture levels retrieved from the satellite data are well correlated with precipitation events: a strong increase in soil moisture is observed, following each significant rainfall event.

#### 4.1.2. Method 2 Validation with Ground Measurements

**Figure 10:**
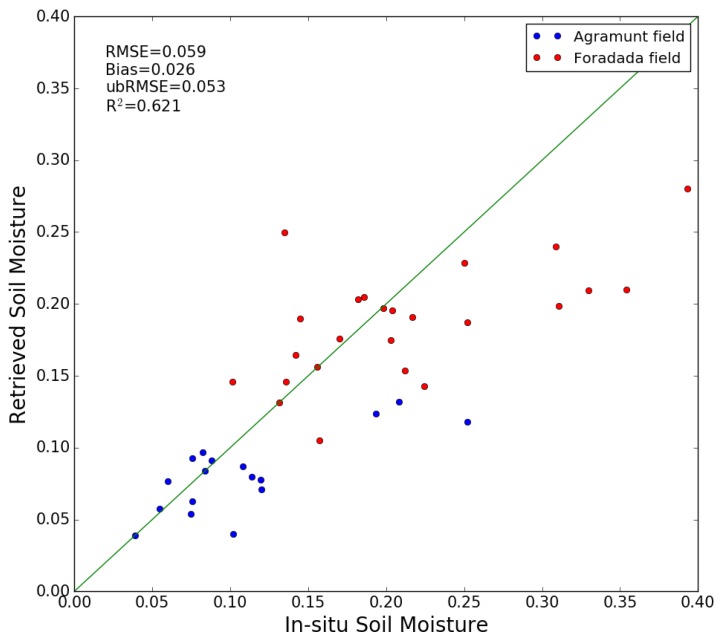
Intercomparison between ground measurements in the two demonstration fields of Agramunt and Foradada and S1 moisture estimations based on method 2.

**Figure 11:**
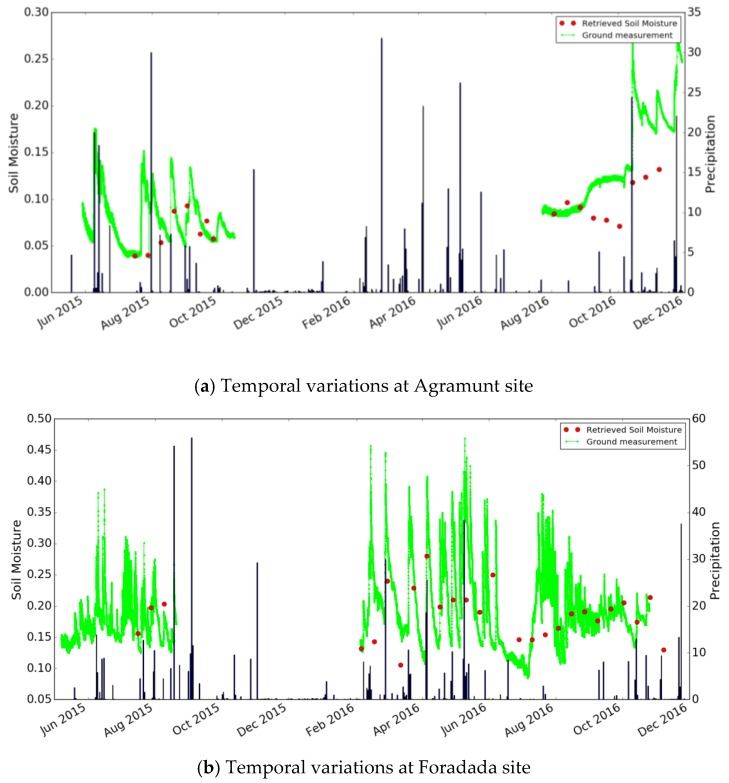
Temporal variations in ground measurements and S1 estimations of soil moisture at the Agramunt site (**a**) and Foradada site (**b**), determined using method 2.

Figure 10 compares the ground measurements with the estimated values of soil moisture obtained with method 2. From this regression, the RMSE in volumetric soil moisture is 0.059 m^3^/m^3^, and the unbiased RMSE is 0.053 m^3^/m^3^. The RMSE is respectively equal to 0.048 m^3^/m^3^ and 0.066 m^3^/m^3^ for Agramunt and Foradada field, with a bias of 0.028 m^3^/m^3^ and 0.026 m^3^/m^3^ separately. The unbiased RMSE is 0.04 m^3^/m^3^ for Agramunt field and 0.06 m^3^/m^3^ for Foradada field. Figure 11 compares the moisture estimations with ground measurements, as a function of time. The soil moisture values retrieved from satellite data are also well correlated with the observed precipitation events, with the soil moisture increasing after each significant rainfall event. As both method1 and method 2 have a relatively high RMSE, the small number of ground measurements and the relatively small size of the radar signal database could explain this high error.

### 4.2. Discussion

**Figure 12:**
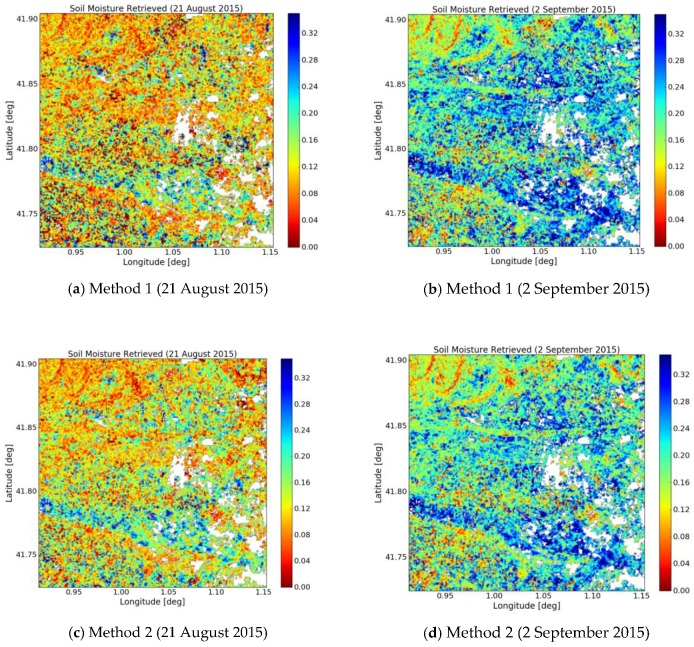
Retrieved soil moisture maps obtained using methods 1 and 2.

The soil moisture at two study sites has been computed and mapped, using Equations (5)–(11) and data produced by Sentinel-1 radar observations. Figure 12 provides two illustrations of moisture mapping, using methods 1 and 2, for two cases: a very dry day (21 August 2015) and a wet day (2 September 2015). All cells with NDVI < 0.1 or NDVI > 0.8, which are associated with water bodies and forests respectively, are masked out. A high similarity is observed between the products obtained with these two methods. We retrieve nearly the same spatial variations in moisture on the two analyzed dates. The first dry case clearly reveals the irrigated fields inside dry area. The second wet case shows high values of soil moisture over most of the observed area.

**Figure 13:**
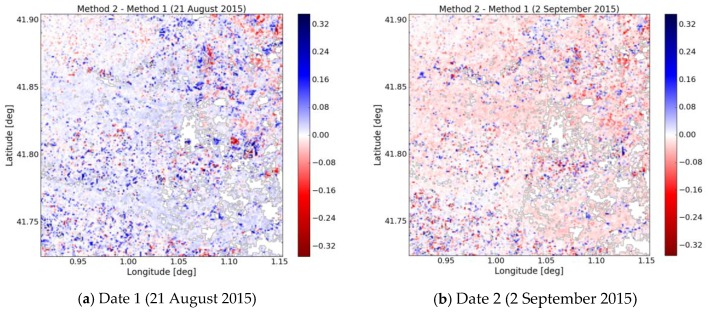
Difference of soil moisture retrieved by method 2 and 1 for date 1 (21 August 2015) and date 2 (2 September 2015).

**Figure 14:**
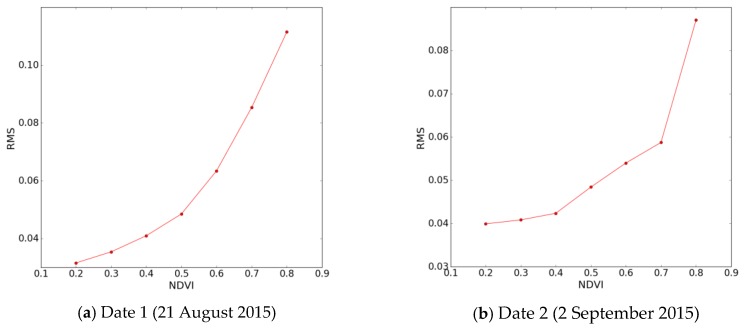
RMS between methods 1 and 2, as a function of NDVI for date 21 August 2015 (**a**) and date 2 September 2015 (**b**).

Figure 13 shows the difference between method 1 and method 2 for date 21 August 2015 and date 2 September 2015. A limited difference is illustrated for the two dates between the two methods. The highest differences correspond to high vegetation density covers. Figure 14 plots the variation in RMSE between methods 1 and 2, as a function of NDVI. This is estimated with a sliding NDVI window, with an NDVI width = 0.2, for NDVI values lying in the range between 0.1 and 0.8. The RMSE can be seen to increase with NDVI. In practice, a high vegetation density can significantly attenuate the signals, thus leading to correspondingly higher errors in soil moisture estimation. Areas with higher vegetation cover are with higher uncertainties. Rapid vegetation change and soil properties such as surface roughness will contribute to uncertainties as well. As change detection approaches, these two methods are very applied operationally since the ground measurements are not prerequisite and that they can be improved with the size of time series.

## 5. Conclusions

In this study, two inversion approaches are developed for the interpretation of high repeat frequency Sentinel-1 radar data in synergy with Sentinel-2 optical data. Change detection techniques in proposed methodologies, are validated with ground measurements carried out in two demonstration fields. The estimated (volumetric) RMS soil moisture errors are approximately 0.087 m^3^/m^3^ for method 1 and 0.059 m^3^/m^3^ for method 2. Both methods are found to predict soil moisture variations that are well correlated with rainfall events. Method 1 models the backscattering difference with the driest value, whereas method 2 is based on the difference between radar signals observed on two consecutive dates, meaning that the radar signals are influenced by much smaller changes in vegetation. Method 2 is found to be more robust than method 1, since it does not require searching for the minimum value in each pixel, which can introduce larger errors under extreme local conditions. The backscattered radar contributions produced by the vegetation are small in the case of method 2. However, as the retrieved value of soil moisture depends on the soil moisture determined at an earlier date with this method, the iterative process can lead to the accumulation of errors. Comparing to other types of inversion algorithms (e.g., NN or calibrated model inversion), both of these methods allow soil moisture to be estimated, with no need for calibrations based on ground measurements, and have led to the production of similar, 100 m resolution soil moisture maps of the study area. SMOS data is used for limiting the maximum soil moisture retrieved by satellite. However, the interest of SMOS (or SMAP or other low-resolution soil moisture sensor) is that it is available globally and needs no local maintenance, which makes our method applicable globally in contrast with methods that require in-situ data such as NN or calibrated model inversion.

These results demonstrate the potential of Sentinel-1 data for the retrieval of 100 m (or even better) resolution soil moisture. Both methods can be applied to any vegetation-covered area for which time-series of SAR and optical data have been recorded. In future, the statistical analysis should be improved by using a larger number of data acquisitions from the S1 time series.

In the present study, data derived from the VV polarization was analyzed, since it is more sensitive to soil conditions. However, Sentinel-1 provides data in both VV and VH polarization modes, and it is planned to include VH polarization analyses in future studies, since this operational mode is highly sensitive to the influence of vegetation, and can be used to discriminate between the effects of vegetation.

## Figures and Tables

**Table 1:** Ground soil moisture measurements in two demonstration fields at Foradada and Agramunt.

Site	Foradada	Agramunt
Coordinates	41.866° N, 1.015° E	41.782° N, 1.089° E
At soil depths in cm	3, 9, 10, 20	5, 10, 20, 40
Period of ground measurements	May–August 2015 February–October 2016	June–October 2015 July–November 2016
Sand, silt, clay in %	41.5, 42.3, 16.2	52.1, 35.3, 12.6
Irrigation method	Sprinklers	Subsurface drippers
Surface soil moisture (min, max) in m^3^/m^3^	(0.08, 0.45)	(0.04, 0.28)
Meteorological station	Baldomar station	Tornabous station

**Table 2:** Sentinel-1 database.

Date	Date	Date	Date	Date	Date
16 July 2015	13 Nov. 2015	05 Feb. 2016	29 Apr. 2016	03 Aug. 2016	26 Oct. 2016
28 July 2015	25 Nov. 2015	17 Feb. 2016	11 May 2016	15 Aug. 2016	07 Nov. 2016
09 Aug. 2015	07 Dec. 2015	29 Feb. 2016	23 May 2016	27 Aug. 2016	19 Nov. 2016
21Aug. 2015	19 Dec. 2015	12 Mar. 2016	04 June 2016	08 Sept. 2016	-
02 Sept. 2015	31 Dec. 2015	24 Mar. 2016	28 June 2016	20 Sept. 2016	-
14 Sept. 2015	12 Jan. 2016	05 Apr. 2016	10 July 2016	02 Oct. 2016	-
26 Sept.2015	24 Jan. 2016	17 Apr. 2016	22 July 2016	14 Oct. 2016	-

**Table 3:** Sentinel-2 database.

Date	Date		Date		Date		Date		Date
06 July 2015	21 Oct. 2015	19 Mar. 2016		21 May 2016		30 July 2016		28 Sept. 2016	
16 July 2015	20 Nov. 2015	22 Mar. 2016		28 May 2016		06 Aug. 2016		05 Oct. 2016	
02 Aug. 2015	30 Nov. 2015	29 Mar. 2016		07 June 2016		09 Aug. 2016		15 Oct. 2016	
05 Aug. 2015	03 Dec. 2015	01 Apr. 2016		10 June 2016		16 Aug. 2016		18 Oct. 2016	
12 Aug. 2015	23 Dec. 2015	08 Apr. 2016		20 June 2016		19 Aug. 2016		25 Oct. 2016	
15 Aug. 2015	30 Dec. 2015	11 Apr. 2016		27 June 2016		26 Aug. 2016		28 Oct. 2016	
22 Aug. 2015	12 Jan. 2016	18 Apr. 2016		30 June 2016		29 Aug. 2016		04 Nov. 2016	
25 Aug. 2015	19 Jan. 2016	28 Apr. 2016		07 July 2016		05 Sept. 2016		07 Nov. 2016	
11 Sept. 2015	29 Jan. 2016	01 May 2016		10 July 2016		08 Sept. 2016		14 Nov. 2016	
14 Sept. 2015	18 Feb. 2016	08 May 2016		17 July 2016		15 Sept. 2016		17 Nov. 2016	
24 Sept. 2015	09 Mar. 2016	11 May 2016		20 July 2016		18 Sept. 2016		24 Nov. 2016	
01 Oct. 2015	12 Mar. 2016	18 May 2016		27 July 2016		25 Sept. 2016		27 Nov. 2016	
